# Intraoperative Radiotherapy Is Not a Better Alternative to Whole Breast Radiotherapy as a Therapeutic Option for Early-Stage Breast Cancer

**DOI:** 10.3389/fonc.2021.737982

**Published:** 2021-12-16

**Authors:** Linwei Wang, Min Sun, Shuailong Yang, Yuanyuan Chen, Tian Li

**Affiliations:** ^1^ Department of Radiation and Medical Oncology, Hubei Key Laboratory of Tumor Biological Behaviors/Hubei Cancer Clinical Study Center, Zhongnan Hospital, Wuhan University, Wuhan, China; ^2^ Department of General Surgery, Taihe Hospital, Hubei University of Medicine, Shiyan, China; ^3^ Department of Radiation and Medical Oncology, The Central Hospital of Wuhan, Wuhan, China; ^4^ School of Basic Medicine, Fourth Military Medical University, Xi’an, China

**Keywords:** breast cancer, intraoperative radiotherapy, whole breast radiotherapy, meta-analysis, therapeutic option

## Abstract

**Objective:**

Intraoperative radiotherapy (IORT) in early-stage breast cancer has been studied over the years. However, it has not been demonstrated whether IORT is more suitable as a therapeutic option for early-stage breast cancer than whole breast radiotherapy (WBRT). Therefore, we performed a meta-analysis to compare the efficacy and safety of IORT to those of WBRT as therapeutic options for early-stage breast cancer patients receiving breast-conserving surgery (INPLASY2020120008).

**Methods:**

PubMed, Embase, and Cochrane Library databases were searched from inception to October 2021. Computerized and manual searches were adopted to identify eligible randomized control trials from online databases. Risk ratio (RR) and 95% confidence intervals (CI) were calculated by random-effect models to assess the relative risk. Potential publication bias was quantified by Begg’s and Egger’s tests.

**Results:**

Based on our inclusion criteria, 10 randomized control trials involving 5,698 patients were included in this meta-analysis. This meta-analysis showed that the IORT group was associated with a higher local recurrence risk (RR = 2.111, 95% CI, 1.130–3.943, *p* = 0.0191), especially in the long-term follow-up subgroup or published after 2020 subgroup or Caucasian subgroup (RR = 2.404, 95% CI, 1.183–4.885, *p* = 0.0154). Subgroup analysis showed that the IORT group had a higher recurrence risk than the WBRT group in the polycentric randomized controlled trial subgroup (RR = 1.213, 95% CI, 1.030–1.428, *p* = 0.0204). Pooled analysis showed that there was no statistically significant difference in overall survival, recurrence-free survival, distant metastasis-free survival, and cancer-specific survival between IORT and WBRT groups. Additionally, the risk of skin toxicity was reduced, but the incidences of fat toxicity, edema, and scar calcification were significantly increased in the patients who underwent IORT in comparison to those who underwent WBRT.

**Conclusion:**

This meta-analysis revealed that IORT was not a better alternative to WBRT. More large-scale and well-designed clinical trials with longer follow-up periods are encouraged to further investigate the value of IORT.

**Systematic Review Registration:**

https://inplasy.com/inplasy-2020-12-0008/.

## Introduction

Breast cancer (BC) is the most common malignant tumor. Globally, it is a leading cause of cancer-associated mortalities among women (1, 2). Advances in screening and treatment technology have revealed that lymph node-negative early-stage BC is highly prevalent in BC patients (3). As a therapeutic option with less psychological and physiological trauma, breast-conserving surgery (BCS) is widely accepted by early-stage BC patients, especially lymph node-negative BC patients (3, 4). Postoperative whole breast radiotherapy (WBRT) is an effective supplement for BCS. Compared to lumpectomy alone, WBRT inhibits the rate of local recurrence by 24.9% (5). Moreover, the distant metastasis rate is decreased after radiotherapy (4, 6). Therefore, BCS followed by radiotherapy has been a standard clinical care (7, 8). However, this therapeutic strategy is associated with long-time treatment course, imprecise positioning, and damage to normal tissues.

Radiotherapeutic approaches have been developed to overcome the problems associated with WBRT. Accelerated partial breast irradiation (APBI) is gradually becoming a surrogate to WBRT, because it can effectively shorten the treatment time to 1–2 weeks, decrease long-term treatment complications, and improve the quality of life (9). APBI can be performed with multiple different methods including external beam radiotherapy, low-dose-rate brachytherapy, high-dose-rate interstitial brachytherapy, single- or multi-lumen balloon intracavitary brachytherapy, and intraoperative radiotherapy (IORT). Various studies have been performed to evaluate the efficacy and safety of the above different techniques for APBI (10, 11). As an important APBI modality, IORT is introduced in BC treatment within a shorter time than other radiotherapeutic techniques. By using dedicated linear accelerators or novel mobile devices, IORT can directly deliver a single radiation dose to the tumor bed in the operating room (12–14). Moreover, IORT improves the accuracy of radiotherapeutic administration to protect normal tissues from damage. Some studies have indicated that IORT can considerably improve breast fibrosis, retraction, and edema; provide good admission for patients; and allow faster recommencement of job and housework (15, 16).

A meta-analysis by Zhang et al. (17) revealed that IORT had fewer side effects, better cosmetic effects, and undifferentiated BC and non-BC mortality rates than those of WBRT. However, the risk of ipsilateral breast tumor recurrence was significantly higher in the IORT group than in the WBRT group (17). Additionally, the studies included in that meta-analysis were limited to two randomized controlled trials (RCTs) and two non-RCTs. The two RCTs were published several years ago and updated in 2020 and 2021. In the present study, we conducted an updated and more comprehensive meta-analysis and subgroup analysis to reveal the prognostic value and adverse effect (AE) association of IORT and WBRT for BC.

## Methods

### Study Protocol

This article was performed according to the Preferred Reporting Items for Systematic Reviews and Meta-Analyses (PRISMA) statement (18). The protocol was registered on INPLASY (INPLASY2020120008) (https://inplasy.com/inplasy-2020-12-0008/).

### Search Strategy

All eligible RCTs that compared IORT to WBRT in BC patients receiving BCS were identified from PubMed, the Cochrane Library, Science Direct, and China Biology Medicine databases up to October 2021. The keywords used include “breast neoplasms,” “breast cancer,” “breast carcinoma,” “radiotherapy,” “radiation,” “intraoperative,” and “IORT.” We manually searched the reference lists of relevant reviews while abstracts from international conferences were also reviewed. Publication languages were limited to English and Chinese.

### Eligibility Criteria

Eligible patients were conformed to the following criteria: 1) histologically confirmed as stage I or II BC patients who underwent BCS; 2) no preoperative anticancer treatments; 3) no other site cancer besides breast; 4) no serious organ (liver, kidney, or heart) dysfunction; and 5) randomly assigned to receive IORT or WBRT. The exclusion criteria were as follows: 1) studies examining IORT as a “boost dose” followed by WBRT; 2) tumor location was not easily accessible by the IORT equipment, such as in the tail of the breast; 3) loss of follow-up rate that was higher than 20%; 4) the ones with shorter follow-up for multiple articles presenting the same clinical trial; and 5) non-RCTs. In this study, the IORT group was defined as patients receiving BCS and IORT while the WBRT group was defined as patients receiving BCS followed by WBRT.

### Data Extraction

Two researchers (YC and MS) independently extracted detailed information regarding the publication year, first author, median follow-up time, radiotherapeutic planning, survival data, and radiotherapeutic-associated AEs from each trial. Discrepancies were resolved by discussions with a third author (TL) to reach a consensus. In this meta-analysis, recurrence or death within 2 years after diagnosis was defined as short-term survival, while that within more than 2 years was defined as long-term survival. Overall survival (OS) was defined as the time from diagnosis or surgery to death due to any cause or last follow-up visit. Recurrence-free survival (RFS) was defined as the time from diagnosis or surgery to any BC recurrence. Locoregional recurrence-free survival (LRFS) was defined as the time from diagnosis or surgery to any recurrence or reappearance of the ipsilateral preserved breast, chest wall, or lymphatic drainage area. Distant metastasis-free survival (DMFS) was defined as the time from diagnosis to or surgery, the date of distant metastasis, or when censored at the latest date. Cancer-specific survival (CSS) was defined as the duration from the date of diagnosis to death due to BC.

The primary end points were LRFS, OS, RFS, DMFS, and CSS. The second end points included seroma, fat toxicity, excellent/good cosmetic outcome, pulmonary fibrosis, edema, skin toxicity, pain, architectural distortion, any retraction, and scar calcification. The outcome measure was assessed independently. For the duplicate or subgroup studies, the most recent and complete data were extracted.

### Quality Assessment

Quality of the enrolled RCTs was independently evaluated by two authors (YC and MS) according to the Cochrane Collaboration’s risk-of-bias tool (19), which included the adequacy of random sequence generation, allocation concealment, blinding of participants and personnel, blinding of outcome assessment, incomplete outcome data, selective outcome reporting, and other bias. Each item was assessed as low, high, and unclear risk of bias. If there was any disagreement, another author (LW) reviewed the materials again to reach a consensus.

### Statistical Analysis

We employed the package meta and metafor in R-3.6.3 (R Core Team, Vienna, Austria, 2020) for statistical analysis. Risk ratios (RR) and 95% confidence interval (CI) were calculated to estimate the relative risk of survival and AEs. Statistical heterogeneity in the included trials was evaluated by the chi-square test and quantified with the I^2^ statistic. The existence of significantly statistical homogeneity was considered unreasonable if *p* < 0.10 or I^2^ > 40%. In this case, the random-effect model was used to estimate the relative risk of the efficacy and safety. Otherwise, the fixed-effect model was used. Potential publication bias was evaluated by Begg’s and Egger’s tests (20, 21). *p* ≤ 0.05 was considered to be statistically significant.

## Results

### Characteristics of Included RCTs

**Figure 1:**
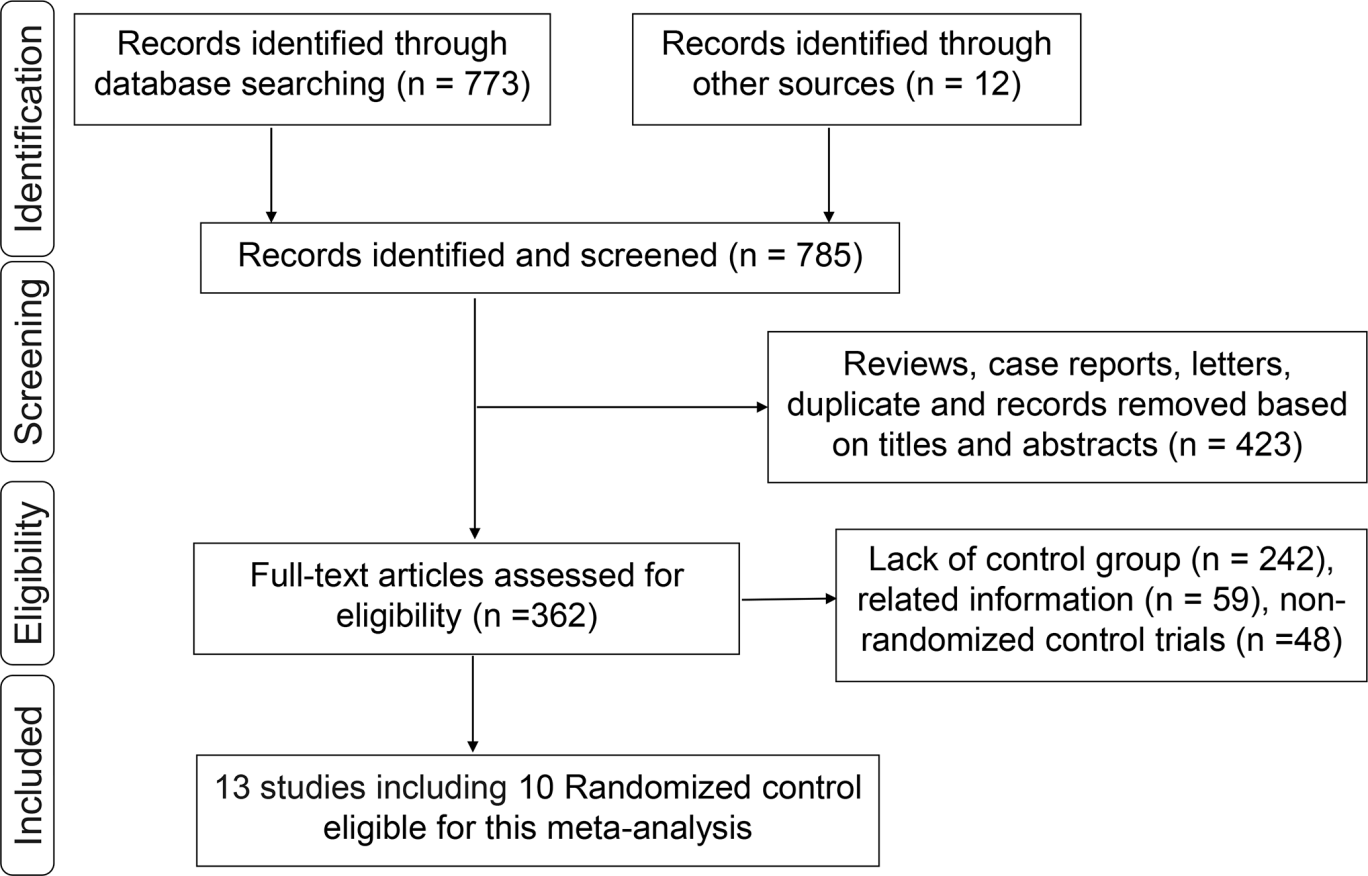
Flowchart of the identification process for eligible studies.

A total of 773 studies were obtained from databases, and 12 studies were obtained from relevant references. According to the inclusion criteria, 13 studies including 10 RCTs were finally identified. Among the 10 RCTs, one multicenter RCT (TARGIT-A trial) was first performed by Vaidya et al. in 2010 (22), then updated in 2014 (23), 2016 (24), and 2020 (25, 26). The latest data were included in this meta-analysis. The study by Rampinelli et al. (27) was excluded from this analysis because it only reported the information of pulmonary fibrosis which had been described by Veronesi et al. (12) and updated in 2021 (28). Six RCTs (24, 29–33) derived from different centers of the TARGIT-A trial were included in this analysis, which evaluated radiotherapy-related AEs different with the TARGIT-A trial. Finally, 13 studies including 10 RCTs (2, 12, 14, 23–26, 28–33) involving 5,698 patients were eligible for this meta-analysis. The median follow-up time ranged from 0.67 to 18.9 years. The screening process was as shown in **Figure 1**. The baseline characteristics of these studies are summarized in **Table 1**.

**Table 1:** Baseline characteristics of the included RCTs.

RCTs	Patients (n)		Treatment planning		IORT device	County	Median follow-up (months)
	IORT	WBRT	IORT	WBRT			
Vaidya (22–24)	1,721	1,730	20 Gy	40–56 Gy with or without a boost of 10–16 Gy, standard tangents	The Intrabeam device (Carl Zeiss Meditec, Oberkochen, Germany)	Multicenter	60.0
Elsberger (29)	61	80	20 Gy	40–56 Gy with or without a boost of 10–16 Gy, standard tangents	The Intrabeam device (Carl Zeiss Meditec, Oberkochen, Germany)	Tayside, Scotland	51.6/61.2
Veronesi & Orecchia (12, 28)	651	654	21 Gy	50 Gy given in 25 fractions using tangential beams, followed by a boost dose of 10 Gy in 5 fractions	NOVAC 7 (Hythesis, Latina, Italy) and Liac (Info and Tech, Rome, Italy)	Multicenter	69.6
Peng (34)	60	60	21 Gy	NA	Linear accelerator	China	8.0–24.0
Xiao (14)	70	70	21 Gy	NA	Linear accelerator	China	8.0–24.0
Engel (30)	27	21	20 Gy	40–56 Gy with or without a boost of 10–16 Gy, standard tangents	The Intrabeam device (Carl Zeiss Meditec, Oberkochen, Germany)	Heidelberg, Germany	51.6
Andersen (33)	126	112	20 Gy	40–56 Gy with or without a boost of 10–16 Gy, standard tangents	The Intrabeam device (Carl Zeiss Meditec, Oberkochen, Germany)	Copenhagen, Denmark	17.4/17.1
Sperk (31)	54	55	20 Gy	40–56 Gy with or without a boost of 10–16 Gy, standard tangents	The Intrabeam device (Carl Zeiss Meditec, Oberkochen, Germany)	Mannheim, Germany	40.0/42.0
Rivera (32)	14	16	20 Gy	40–56 Gy with or without a boost of 10–16 Gy, standard tangents	The Intrabeam device (Carl Zeiss Meditec, Oberkochen, Germany)	Los Angeles, California	48.0
Corica (24)	60	66	16–33 Gy	45–50.4 Gy in 25–28 fractions	The Intrabeam device (Carl Zeiss Meditec, Oberkochen, Germany)	Western Australia	47

RCTs, randomized controlled trials; IORT, intraoperative radiotherapy; WBRT, whole breast radiotherapy; NA, not available.

### Methodological Assessment

The description of random sequence generation was definite in all RCTs, 8 of which (2, 12, 23, 24, 29–33) used allocation concealment and was blinded in outcome assessment, and 2 of which (14) did not mention the details of the allocation method and blinding. Participant blinding and personnel assessment were unclear in these trials. Notably, it was not always feasible to perform participant blinding because of surgical trial specificity. No reporting bias was observed in the included RCTs. Other bias might exist in the trial by Rivera et al. (32) due to the small sample size. Risk bias is shown in **Supplementary Figure 1**.

### Primary Analysis of Local Recurrence Analysis for IORT Versus WBRT

**Figure 2:**
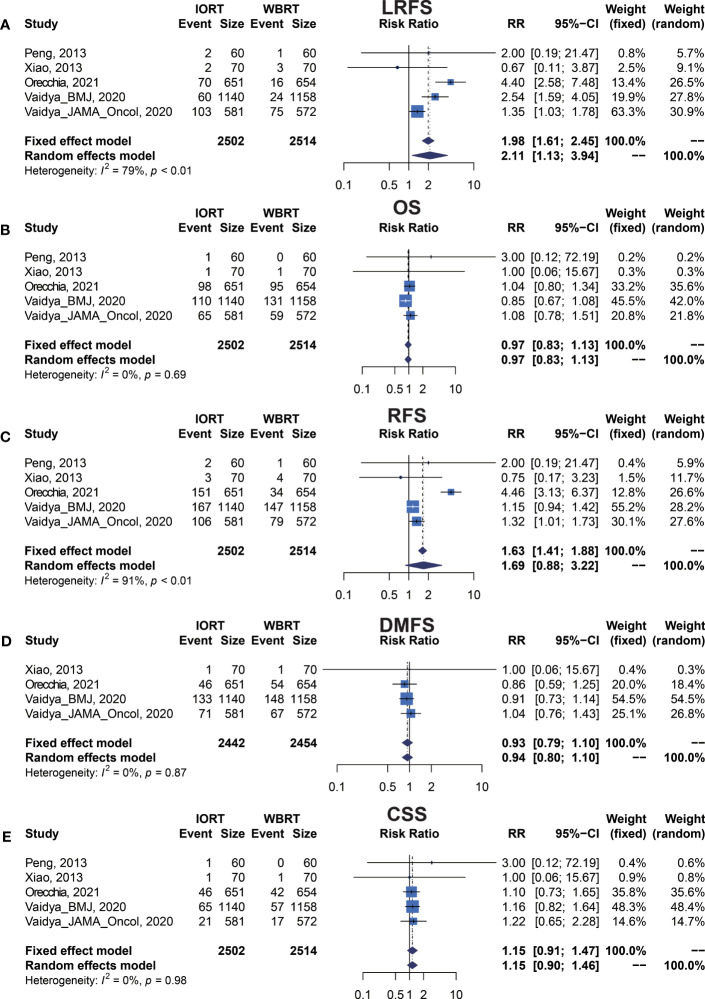
Forest plots of main survival outcomes. **(A)** LRFS. **(B)** OS. **(C)** RFS. **(D)** DMFS. **(E)** CSS.

**Figure 3:**
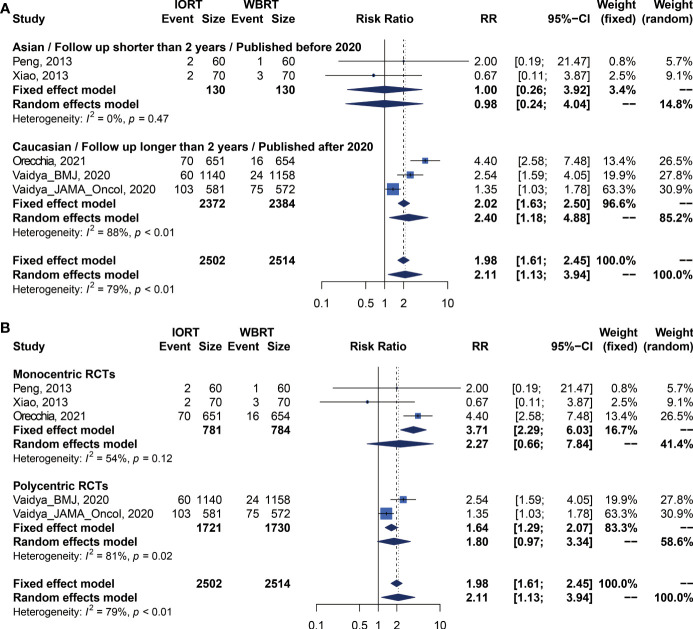
Subgroup analysis for LRFS in breast cancer patients with IORT vs. EBRT. **(A)** Asian/Follow-up shorter than 2 years/Published before 2020 subgroup and Caucasian/Follow-up longer than 2 years/Published after 2020 subgroup. **(B)** Monocentric RCT and polycentric RCT subgroups.

We performed meta-analysis of LRFS, OS, RFS, DMFS, and CSS for IORT versus WBRT (**Figure 2**). The results and analysis of publication bias are presented in **Table 2**. As for the LRFS, there were 5 trials involving 5,016 patients reporting the short-term (14, 34) and long-term (2, 12, 23, 25, 26) local recurrence. Pooled analysis showed that the IORT group was associated with a higher local recurrence risk (RR = 2.111, 95% CI, 1.130–3.943, *p* = 0.0191). Subgroup analysis showed that there was no statistical difference between IORT and WBRT groups in the short-term follow-up group or published before 2020 group or Asian group local recurrence (RR = 1.000, 95% CI, 0.255–3.916, *p* = 1.000). However, the local recurrence risk of the long-term follow-up subgroup or published after 2020 subgroup or Caucasian subgroup in the IORT group was higher than that in the WBRT group (RR = 2.404, 95% CI, 1.183–4.885, *p* = 0.0154) (**Figure 3A** and **Table 3**). No significant difference was found in the monocentric or polycentric RCT subgroup between IORT and WBRT groups (**Figure 3B** and **Table 3**).

**Table 2:** Survival outcomes of pooled estimations of OS, RFS, LRFS, DMFS, and CSS in breast cancer patients with IORT and WBRT.

Outcome	No. of trials (patients)	RR (95% CI)Fixed-effect estimate	p value of fixed-effect model	RR (95% CI)Random-effect estimate	p value of random-effect model	Heterogeneity I^2^ (%)	p value of heterogeneity	p value of Egger’s test	p value of Begg’s test
LRFS	5 (5016)	1.984 (1.609–2.447)	<0.0001	***2.111 (1.130–3.943)***	***0.0191***	78.6%	0.0009	0.7216	0.6242
OS	5 (5016)	0.966 (0.827–1.129)	0.6649	0.967 (0.828–1.129)	0.6705	0.0%	0.6869	0.4383	0.3272
RFS	5 (5016)	1.626 (1.409–1.876)	<0.0001	1.687 (0.884–3.219)	0.1127	91.1%	<0.0001	0.7468	0.1416
DMFS	4 (4896)	0.934 (0.795–1.099)	0.4120	0.935 (0.795–1.100)	0.4177	0.0%	0.8663	0.9333	1.0000
CSS	5 (5016)	1.153 (0.906–1.466)	0.2477	1.150 (0.904–1.464)	0.2540	0.0%	0.9793	0.3080	0.3272

RR, relative risk; CI, confidence interval; OS, overall survival; RFS, recurrence-free survival; LRFS, local recurrence-free survival; DMFS, distant metastasis-free survival; CSS, cancer-specific survival.

I^2^: index for assessing heterogeneity; value ≥40% indicates a moderate to high heterogeneity.

Egger’s test: p value of Egger’s regression for asymmetry assessment.

Begg’s test: p value of Begg and Mazumdar rank correlation test for asymmetry assessment.

Bold italics indicate statistically significant values (p < 0.05).

**Table 3:** Subgroup analysis for survival outcomes of OS, RFS, LRFS, DMFS, and CSS in breast cancer patients with IORT and WBRT.

Outcome	Subgroups	No. of trials	RR (95% CI)	p value of fixed-effect model	RR (95% CI)	p value of random-effect model	I^2^	Heterogeneity p	p between subgroupRandom-effect estimate
			Fixed-effect estimate		Random-effect estimate				
LRFS	Before 2020/Asian/Follow-up time shorter than 2 years	2	1.000 [0.255; 3.916]	1	0.984 [0.240; 4.042]	0.9820	0.00%	0.4658	0.2681
	After 2020/Caucasian/Follow-up time longer than 2 years	3	2.018 [1.632; 2.496]	<0.0001	***2.404 [1.183; 4.885]***	***0.0154***	88.47%	0.0002	
	Monocentric	3	3.715 [2.289; 6.028]	<0.0001	2.272 [0.658; 7.837]	0.1941	53.62%	0.1158	0.7417
	Polycentric	2	1.637 [1.294; 2.070]	<0.0001	1.800 [0.970; 3.341]	0.0625	81.04%	0.0216	
OS	Before 2020/Asian/Follow-up time shorter than 2 years	2	1.667 [0.223; 12.448]	0.6185	1.601 [0.200; 12.826]	0.6578	0.00%	0.6077	0.6340
	After 2020/Caucasian/Follow-up time longer than 2 years	3	0.963 [0.824; 1.125]	0.6313	0.964 [0.825; 1.127]	0.6456	0.00%	0.4111	
	Monocentric	3	1.046 [0.808; 1.354]	0.7321	1.043 [0.806; 1.351]	0.7479	0.00%	0.8076	0.5314
	Polycentric	2	0.926 [0.762; 1.124]	0.4360	0.934 [0.744; 1.174]	0.5611	24.06%	0.2512	
RFS	Before 2020/Asian/Follow-up time shorter than 2 years	2	1.000 [0.297; 3.367]	1.0000	0.982 [0.283; 3.404]	0.9767	0.00%	0.4899	0.3810
	After 2020/Caucasian/Follow-up time longer than 2 years	3	1.638 [1.418; 1.892]	<0.0001	1.871 [0.900; 3.888]	0.0933	95.46%	<0.0001	
	Monocentric	3	4.017 [2.871; 5.621]	<0.0001	2.211 [0.620; 7.885]	0.2214	65.17%	0.0566	0.3591
	Polycentric	2	***1.213 [1.030; 1.428]***	***0.0204***	1.214 [1.031; 1.429]	0.0201	0.00%	0.4327	
DMFS	Before 2020/Asian/Follow-up time shorter than 2 years	1	1.000 [0.064; 15.673]	1.0000	1.000 [0.064; 15.673]	1.0000	NA %	1.0000	0.9619
	After 2020/Caucasian/Follow-up time longer than 2 years	3	0.934 [0.794; 1.099]	0.4110	0.935 [0.795; 1.100]	0.4169	0.00%	0.6953	
	Monocentric	2	0.858 [0.590; 1.248]	0.4242	0.858 [0.590; 1.248]	0.4237	0.00%	0.9125	0.6182
	Polycentric	2	0.954 [0.797; 1.142]	0.6068	0.954 [0.797; 1.142]	0.6066	0.00%	0.4936	
CSS	Before 2020/Asian/Follow-up time shorter than 2 years	2	1.667 [0.223; 12.448]	0.6185	1.601 [0.200; 12.826]	0.6578	0.00%	0.6077	0.7543
	After 2020/Caucasian/Follow-up time longer than 2 years	3	1.146 [0.899; 1.460]	0.2711	1.145 [0.899; 1.460]	0.2728	0.00%	0.9622	
	Monocentric	3	1.120 [0.754; 1.664]	0.5751	1.115 [0.750; 1.658]	0.5895	0.00%	0.8259	0.8470
	Polycentric	2	1.172 [0.865; 1.587]	0.3055	1.172 [0.865; 1.587]	0.3062	0.00%	0.8942	

RR, relative risk; CI, confidence interval; OS, overall survival; RFS, recurrence-free survival; LRFS, local recurrence-free survival; DMFS, distant metastasis-free survival; CSS, cancer-specific survival.

I^2^: index for assessing heterogeneity; value ≥40% indicates a moderate to high heterogeneity.

Bold italics indicate statistically significant values (p < 0.05).

### Primary Analysis of OS for IORT Versus WBRT

**Figure 4:**
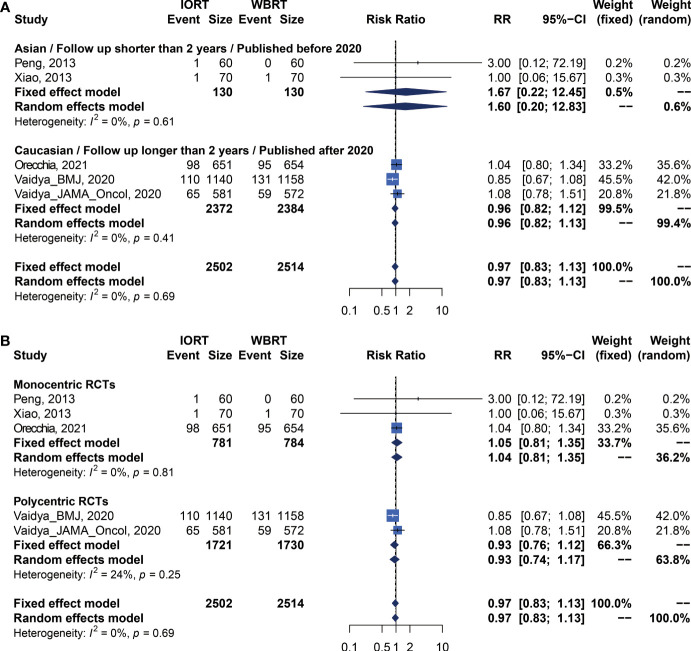
Subgroup analysis for OS in breast cancer patients with IORT vs. EBRT. **(A)** Asian/Follow-up shorter than 2 years/Published before 2020 subgroup and Caucasian/Follow-up longer than 2 years/Published after 2020 subgroup. **(B)** Monocentric RCT and polycentric RCT subgroups.

Data on OS were available in 5 RCTs involving 5,016 patients (2, 12, 14, 23, 25, 26, 28, 34). Pooled analysis showed there was no statistical difference in OS between IORT and WBRT groups (RR = 0.966, 95% CI, 0.827–1.129, *p* = 0.6649). Subgroup analysis showed that the difference in follow-up time, publication year, race, or RCT type between the two groups had no statistical significance (**Figure 4** and **Table 3**).

### Primary Analysis of RFS for IORT Versus WBRT

**Figure 5:**
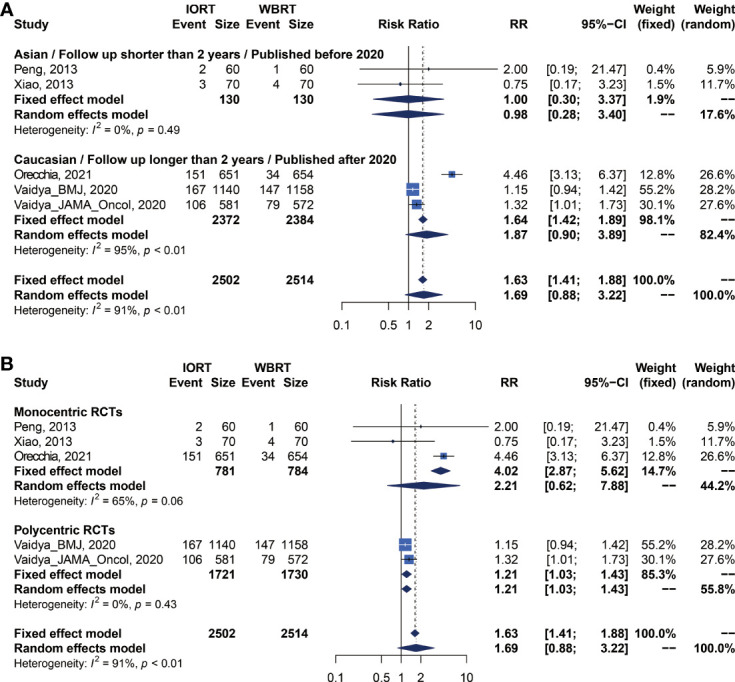
Subgroup analysis for RFS in breast cancer patients with IORT vs. EBRT. **(A)** Asian/Follow-up shorter than 2 years/Published before 2020 subgroup and Caucasian/Follow-up longer than 2 years/Published after 2020 subgroup. **(B)** Monocentric RCT and polycentric RCT subgroups.

There were 5 trials involving 5,016 patients reporting RFS. Pooled analysis indicated that there was no significant difference between the IORT and WBRT groups in RFS (RR = 1.687, 95% CI, 0.884–3.219, *p* = 0.1127, **Table 2**). Subgroup analysis showed that the IORT group had a higher recurrence risk than the WBRT group in the polycentric RCT subgroup without between-study heterogeneity (I^2^ = 0%) (RR = 1.213, 95% CI, 1.030–1.428, *p* = 0.0204, **Figure 5** and **Table 3**).

### DMFS and CCS Analysis for IORT Versus WBRT

**Figure 6:**
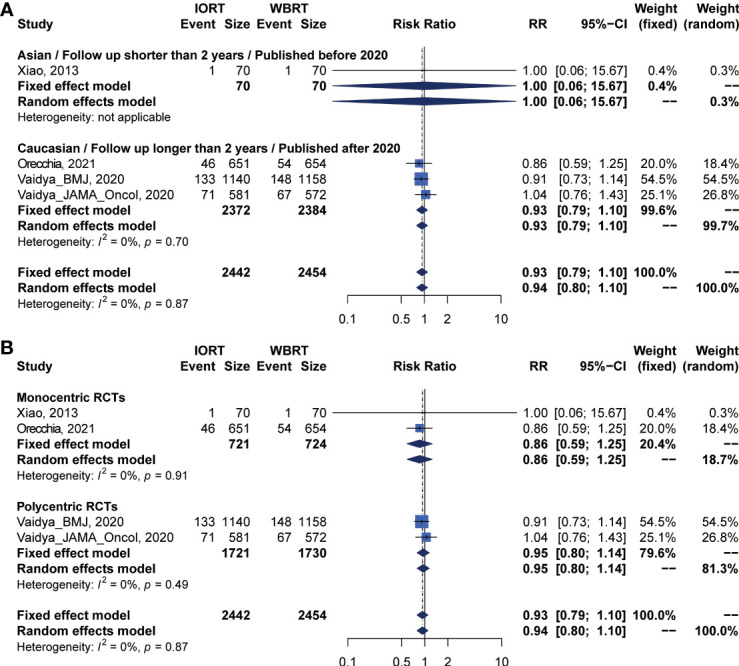
Subgroup analysis for DMFS in breast cancer patients with IORT vs. EBRT. **(A)** Asian/Follow-up shorter than 2 years/Published before 2020 subgroup and Caucasian/Follow-up longer than 2 years/Published after 2020 subgroup. **(B)** Monocentric RCT and polycentric RCT subgroups.

**Figure 7:**
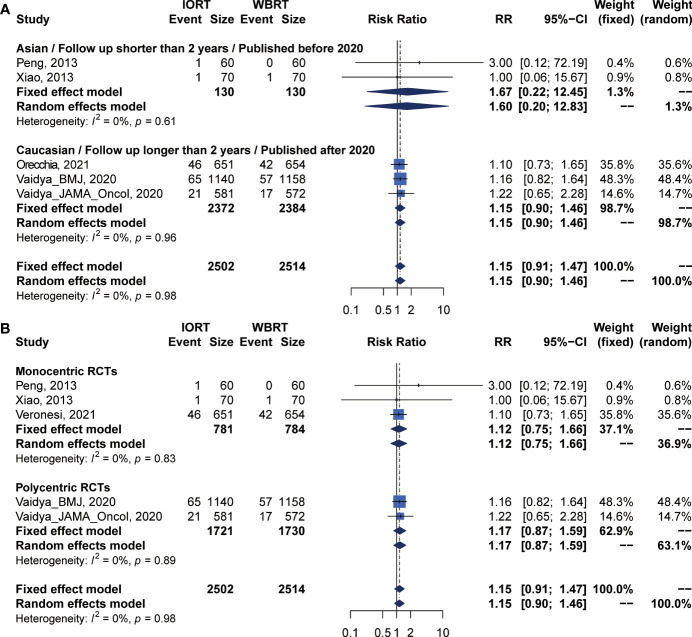
Subgroup analysis for CSS in breast cancer patients with IORT vs. EBRT. **(A)** Asian/Follow-up shorter than 2 years/Published before 2020 subgroup and Caucasian/Follow-up longer than 2 years/Published after 2020 subgroup. **(B)** Monocentric RCT and polycentric RCT subgroups.

Pooled analysis showed there was no statistical difference in DMFS and CCS between IORT and WBRT groups (**Table 2** and **Figure 2**). Subgroup analysis based on the follow-up time, publication year, race, or RCT subgroup showed that the difference between the two groups had no statistical significance (**Figures 6**, **7** and **Table 3**).

### Radiotherapy-Related AE Analysis for IORT Versus WBRT

Detailed information on radiotherapy-related AEs is shown in **Table 4**. There was no statistical difference in architectural distortion (*p* = 0.7726), any retraction (*p* = 0.1437), and pain (*p* = 0.2730) between the two groups. The risk of skin toxicity (12, 23) (RR = 0.275, 95% CI, 0.156–0.486, *p* < 0.0001, **Table 4**) was significantly lower in the IORT group than in the WBRT group. However, a pooled analysis of 5 trials (14, 29, 30, 32, 34) showed that the fat toxicity incidence in the IORT group was 3.106 times higher than that in the WBRT group (RR = 4.106, 95% CI, 1.951–8.638, *p* = 0.0002). There were 3 and 2 trials on scar calcification (29, 30, 32) and edema (14, 34), respectively, which showed that the differences between IORT and WBRT groups had statistical significance (RR = 2.328, 95% CI, 1.193–4.542, *p* = 0.0132; RR = 3.4, 95% CI, 1.290–8.959, *p* = 0.0133). There was no statistical difference in other AEs including seroma (*p* = 0.2664), pulmonary fibrosis (*p* = 0.31), and short-term excellent/good cosmetic outcomes (*p* = 0.1313) between IORT and WBRT groups (**Table 4**).

**Table 4:** Information of radiotherapy-related AEs.

Specific AEs	No. of trials (patients)	RR (95% CI)	p value of Fixed-effect model	RR (95% CI)	p value of Random-effect model	Heterogeneity I^2^ (%)	p value of heterogeneity	p value of Egger’s test	p value of Begg’s test
		Fixed-effect estimate		Random-effect estimate					
Fat toxicity	5 (469)	***4.106 (1.951–8.638)***	***0.0002***	3.346 (1.628–6.876)	0.0010	0.0%	0.7047	0.0399	0.6242
Edema	2 (260)	***3.400 (1.290–8.959)***	***0.0133***	3.108 (0.873–11.069)	0.0801	37.2%	0.2068	NA	NA
Skin toxicity	2 (4327)	***0.275 (0.156–0.486)***	***<0.0001***	0.275 (0.156–0.485)	<0.0001	0.0%	0.8110	NA	NA
Scar calcification	3 (209)	***2.328 (1.193–4.542)***	***0.0132***	2.387 (1.211–4.707)	0.0120	0.0%	0.5417	0.0975	0.1172
Excellent/good cosmetic outcome	3 (386)	1.230 (1.089–1.388)	0.0008	1.225 (0.941–1.594)	0.1313	78.7%	0.0092	0.3950	0.6015
Pulmonary fibrosis	2 (287)	0.254 (0.144–0.447)	<0.0001	0.295 (0.028–3.115)	0.3100	92.6%	0.0002	NA	NA
Seroma	2 (3489)	2.315 (0.527–10.176)	0.2664	2.306 (0.519–10.252)	0.2725	0.0%	0.7391	NA	NA
Pain	2 (347)	0.819 (0.574–1.170)	0.2730	0.848 (0.524–1.372)	0.5012	29.4%	0.2341	NA	NA
Architectural distortion	3 (209)	1.085 (0.899–1.311)	0.3948	1.038 (0.806–1.336)	0.7726	48.5%	0.1433	0.2143	0.1172
Any retraction	2 (139)	1.390 (0.894–2.163)	0.1437	1.338 (0.850–2.105)	0.2082	5.5%	0.3036	NA	NA

RR, relative risk; CI, confidence interval; NA, not available..

I^2^: index for assessing heterogeneity; value ≥40% indicates a moderate to high heterogeneity.

Egger’s test: p value of Egger’s regression for asymmetry assessment.

Begg’s test: p value of Begg and Mazumdar rank correlation test for asymmetry assessment.

Bold italics indicate statistically significant values (p < 0.05).

### Meta-Regression Analysis of Heterogeneity for Survival Parameters and Publication Bias

We performed a meta-regression to explore the source of main survival parameters (**Supplementary Table 1**). All potential factors could not significantly explain heterogeneity in the meta-analyses of survival outcomes in the *post-hoc* analysis, with the exception of follow-up time and negative lymph node rate. Meta-regression analysis demonstrated a statistically significant correlation between follow-up time and RFS (*p* = 0.0133). From the meta-regression result, we conducted a subgroup analysis with groups of short- or long-term follow-up patients (**Figures 5A, B**).

**Figure 8:**
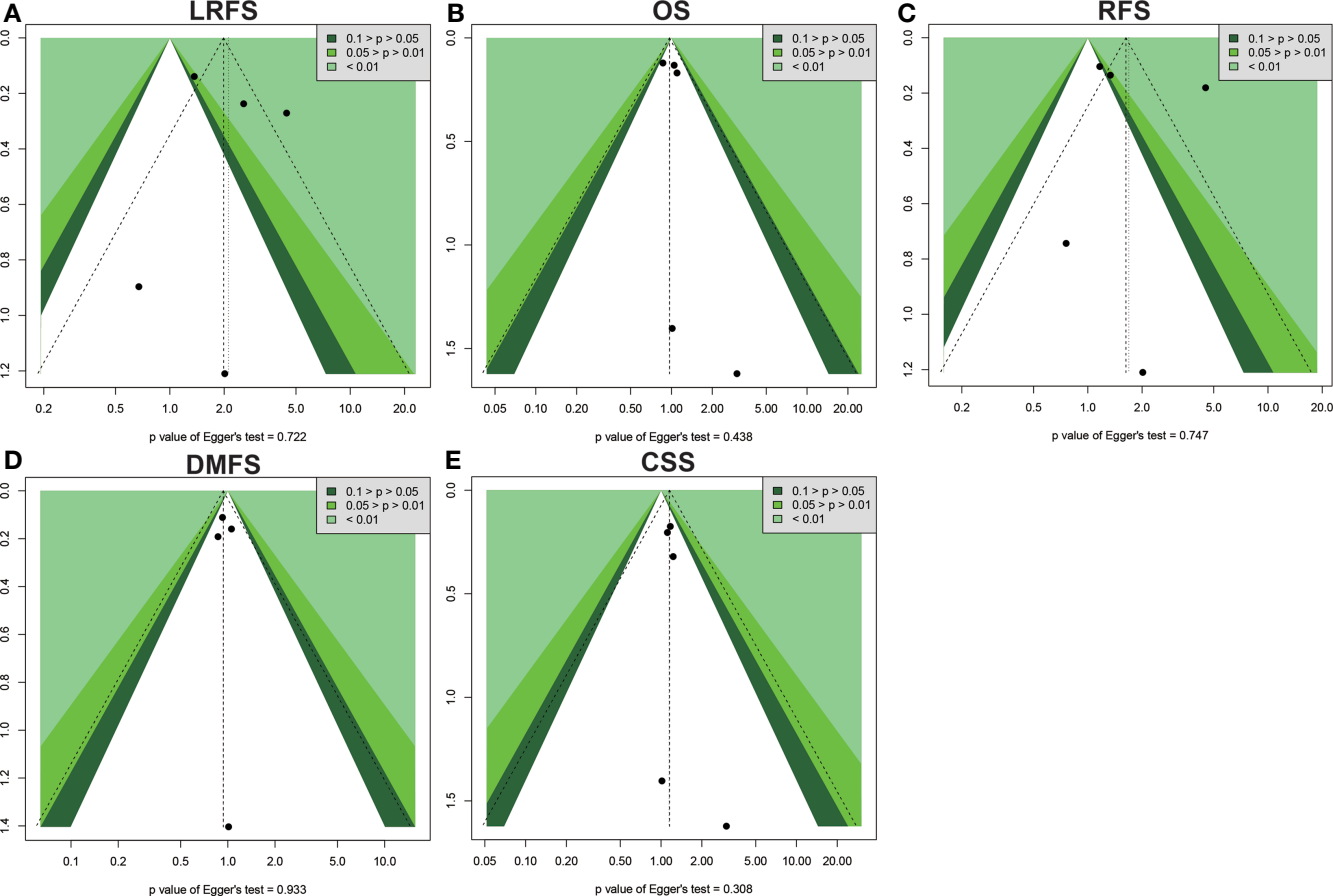
Funnel plot for publication bias in the survival outcomes. **(A)** LRFS. **(B)** OS. **(C)** RFS. **(D)** DMFS. **(E)** CSS.

Begg’s and Egger’s tests were applied to examine the publication bias of main survival parameters and AEs (**Tables 2**, **4**). It was found that there was no obvious publication bias in LRFS, OS, RFS, DMFS, and CSS (**Figure 8**). In regard to fat toxicity, we found that Egger’s regression yielded a potential publication bias (Begg’s test *p* = 0.6242, Egger’s test *p* = 0.0399).

## Discussion

The pooling effect size of this meta-analysis revealed that IORT was not a superior alternative to WBRT in the routine treatment of early-stage BC patients subjected to BCS. This is attributed to the poor local recurrence in the IORT group, especially in the Caucasian population or long-term follow-up subgroup. Our current study indicated that the IORT group had a higher recurrence risk than the WBRT group in the polycentric RCT subgroup. Additionally, the risk of skin toxicity was reduced, but risks of fat toxicity, edema, and scar calcification were significantly increased in the patients who underwent IORT in comparison to those who underwent WBRT.

As an essential adjuvant treatment method, radiotherapy plays a very pivotal role in BC comprehensive therapy. WBRT is a standard radiotherapeutic strategy for early-stage BC patients subjected to BCS. It can significantly improve survival outcomes (6, 35). However, some limitations are associated with the clinical applications of WBRT. Firstly, WBRT should be initiated as soon as after surgery as is practical (36). Postoperative radiotherapy delays have been steadily increasing since the mid-1980s in the UK and other countries (37), which may increase the risk of local recurrence (38). During this intermittent period, the proliferation of residual subclinical lesions may increase the local recurrence rate (38, 39). Simultaneously, tissue repair and anatomical structural alterations during this period could lead to inaccuracies of the irradiation target position (40). Secondly, trials such as the PRIME II trial and the Cancer and Leukemia Group B (CALGB)-9343 trial allow the omission of WBRT which does not affect the 10-year survival rate in low-risk elderly patients (41–46). Thirdly, WBRT may be correlated with AEs to the nearby normal tissue (e.g., skin, lungs, and heart), which affects patients’ regular treatment and breast cosmetic outcome.

Due to these limitations, the IORT technique is introduced as a possible alternative to the conventional WBRT. As a single intensive irradiation dose, IORT can improve the accuracy of radiotherapeutic positioning, shorten the radiotherapeutic course duration, and protect normal tissues in BC patients (47). In addition, IORT is performed during surgery, which could overcome the proliferation of subclinical lesions during the intermission between BCS and radiotherapy. More importantly, IORT can kill cancer stem cells and destroy the tumor microenvironment by enhancing the ability of immune cells to recognize and kill tumors or damage the microvasculature (48–50).

It has not been demonstrated whether IORT is better than WBRT in terms of efficacy and safety. Two large prospective RCTs revealed that the risks of 5-year local recurrence and overall recurrence are significantly higher in the IORT group than in the WBRT group (12, 22–26). A meta-analysis by Zhang et al. (17) showed that ipsilateral breast tumor recurrence was significantly higher in IORT patients. Meanwhile, the differences between the two groups in pooled overall mortality, BC mortality, non-BC mortality and distant metastasis between IORT and WBRT groups had no statistical significance. The findings of Zhang et al. (17) should be interpreted with caution, because 2 of 4 studies were non-RCTs with high bias risks. There was no high-quality meta-analysis comparing the clinical efficacies of IORT and WBRT. Therefore, our meta-analysis based on RCTs was performed to compare the clinical efficacies and safety of IORT with WBRT.

Survival information, the primary prognostic predictor, was adopted to compare the efficacy of IORT and WBRT in this meta-analysis. This meta-analysis showed that there was no statistically significant difference in OS, RFS, DMFS, and CSS between the IORT and WBRT groups. Meanwhile, LFRS or long-term local recurrence in the IORT group was significantly poorer than that in the WBRT group (12, 23). These findings contrast with those of Vaidya et al. who found no statistically significant difference for local recurrence-free survival with long term follow-up (23, 25, 26). It should be noted that some non-therapeutic factors such as tumor size, lymph node stage, histological type, and hormone receptor status might influence patients’ prognosis. A recent study exploring eligible criteria for IORT showed that almost half of T0–T2 patients without lymph node metastasis could be eligible for IORT with expected 5-year free local recurrence rates of 96.6%–98.6% (51). It may be a feasible choice for patients with IORT who have a low risk of local recurrence. Therefore, patients should be carefully selected according to an appropriate criterion before being enrolled in the IORT or WBRT group (52–55). Furthermore, a longer follow-up is needed to obtain the precise results in the IORT and WBRT groups.

Safety is also an important parameter for evaluating therapeutic efficacy. The IORT group had lower incidence of skin toxicity compared to the WBRT group. This was attributed to the single intensive irradiation dose that was directly delivered to the surgical margin, which avoids normal tissue damage. However, the incidences of acute AEs including edema, fat toxicity, and scar calcification in the IORT group were significantly higher than those in the WBRT group. This may be because some non-target tissues nearby the incision received a high irradiation dose in one exposure. There was no statistical difference in other AEs such as pulmonary fibrosis, pain, seroma, architectural distortion, retraction, and excellent/good cosmetic outcomes between the two groups. As a whole, AEs were acceptable and manageable in both two groups.

Based on the above findings, IORT is not a superior alternative for routine WBRT in clinical practice. However, the convenience and therapeutic cost should also be considered in clinical practice. Patients subjected to the traditional WBRT have to undergo radiotherapy for as long as several weeks. IORT can be delivered as a single intensive dose of irradiation during BCS in the operating room, which bypasses the risk of not completing the prescribed radiotherapeutic course (47). Therefore, therapeutic compliance for patients in the IORT group is better than that in the WBRT group. Moreover, Welzel et al. (56) found that patients who received IORT had a comparable quality of life, fewer breast symptoms, less pain, and fewer body image concerns. Furthermore, IORT is a cost-effective option (57). Therefore, for patients in rural communities, IORT might be an alternative choice, because these women tend to be older and live farther from therapeutic centers (58).

Our study has several limitations. Firstly, although Begg’s and Egger’s tests showed no publication bias in survival outcomes, the studies included in this meta-analysis were performed by different investigators in different institutions. Therefore, potential publication bias may still exist. Secondly, due to equipment and technological constraints, studies on IORT have not been extensively applied in clinical practice. Thirdly, due to the limited number of RCTs, it was difficult to make a definitive assessment of the efficacy and safety of WBRT and IORT. Fourthly, this manuscript is a study-level meta-analysis, rather than patient-level. Some non-therapeutic factors or clinical heterogeneities such as tumor size, lymph node stage, histological type, and hormone receptor status might impact on BC patients’ survival outcomes. Fifthly, the study of Veronesi et al. enrolled BC patients who were not completely suitable for IORT (8, 12, 28). Therefore, the results must be interpreted with caution. It is necessary for more meta-analyses to use high-quality studies when investigating the clinical efficacy and safety of IORT versus WBRT in early-stage BC patients receiving BCS based on available RCTs.

In conclusion, IORT had a higher risk of local recurrence than WBRT. IORT is not a better alternative to WBRT as a therapeutic option for BC. Therefore, IORT should be used in conjunction with the prudent selection of suitable patients with a low risk of local recurrence. Large sample RCTs with long-term follow-up are encouraged to further confirm the efficacy and safety of IORT versus WBRT.

## Data Availability Statement

The original contributions presented in the study are included in the article/**Supplementary Material**. Further inquiries can be directed to the corresponding authors.

## Author Contributions

LW: writing. MS and SY: data analysis. YC and TL: idea and revision. All authors contributed to the article and approved the submitted version.

## Funding

This research was supported by the National Natural Science Foundation of China (81702901, 81701768, 81902498), Natural Science Foundation of Hubei Province of China (2019CFB177), Natural Science Foundation of Hubei Provincial Department of Education (Q20182105), Chen Xiao-ping Foundation for the Development of Science and Technology of Hubei Province (CXPJJH11800001-2018333), and Innovation and Entrepreneurship Training Program (201810929005, 201810929009, 201810929068, 201813249010, S201910929009, and S201910929045).

## Conflict of Interest

The authors declare that the research was conducted in the absence of any commercial or financial relationships that could be construed as a potential conflict of interest.

## Publisher’s Note

All claims expressed in this article are solely those of the authors and do not necessarily represent those of their affiliated organizations, or those of the publisher, the editors and the reviewers. Any product that may be evaluated in this article, or claim that may be made by its manufacturer, is not guaranteed or endorsed by the publisher.

## Supplementary Material

Supplementary Figure 1 Risk of bias for all included randomized controlled trials.

Supplementary Table 1 Meta-regression analysis of heterogeneity for survival outcomes.

## Abbreviations

AEs, adverse effects; BC, breast cancer; BCS, breast-conserving surgery; CI, confidence intervals; IORT, intraoperative radiotherapy; RCTs, randomized control trials; RR, risk ratio; WBRT, whole breast radiotherapy; OS, overall survival; APBI, accelerated partial breast irradiation; RFS, recurrence-free survival; LRFS, locoregional recurrence-free survival; DMFS, distant metastasis-free survival; CSS, cancer-specific survival.
