# Supplementary figures and images for: Intraoperative Radiotherapy Is Not a Better Alternative to Whole Breast Radiotherapy as a Therapeutic Option for Early-Stage Breast Cancer
Source: Front Oncol. 2021 Dec 16;11:737982. doi: 10.3389/fonc.2021.737982 (PMC8716392; doi:10.3389/fonc.2021.737982)

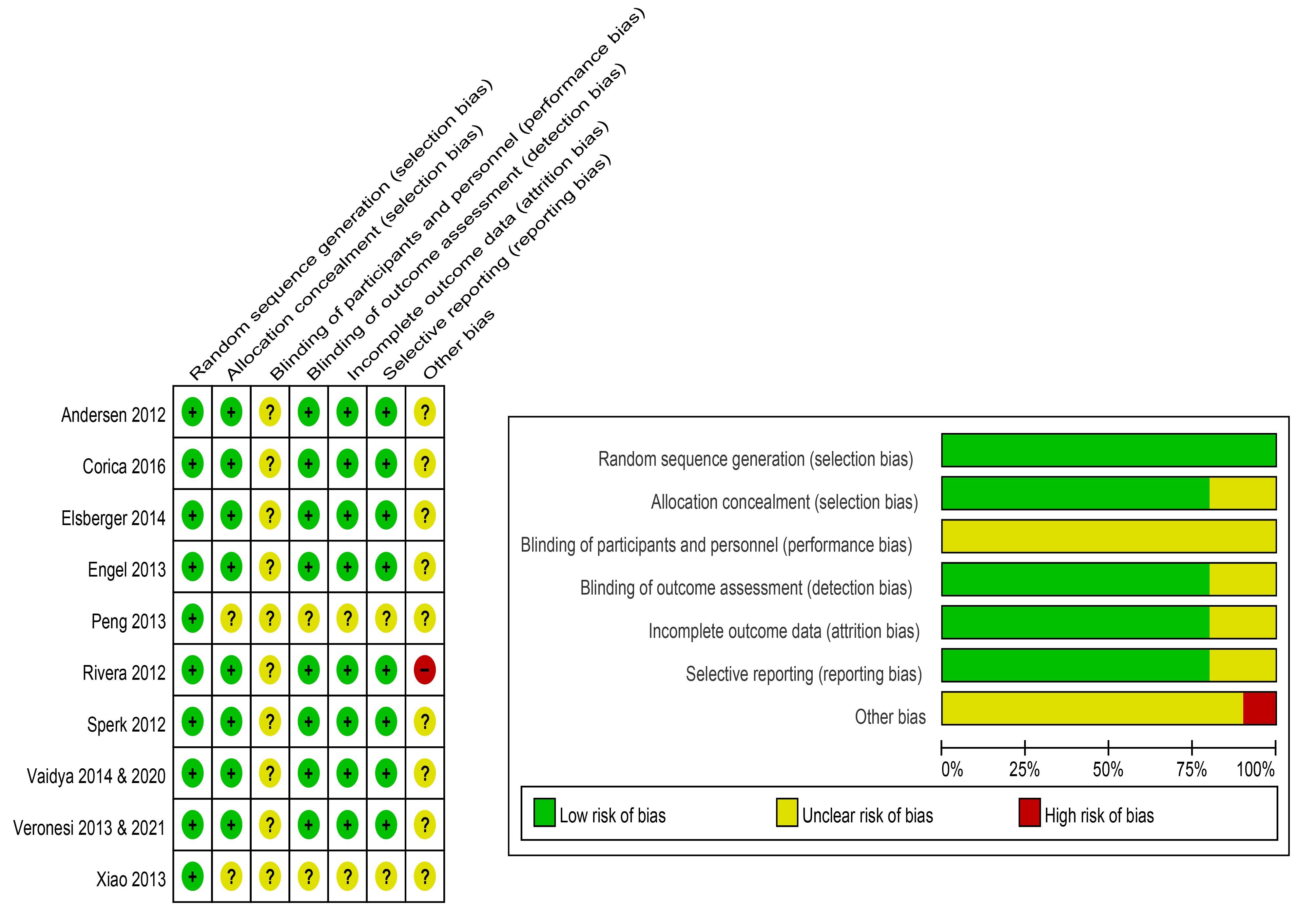

Supplement: Supplementary Figure 1 — Risk of bias for all included randomized controlled trials. [file Image_1.tif]
