# Supplementary material for: Intraoperative Radiotherapy Is Not a Better Alternative to Whole Breast Radiotherapy as a Therapeutic Option for Early-Stage Breast Cancer
Source: Front Oncol. 2021 Dec 16;11:737982. doi: 10.3389/fonc.2021.737982 (PMC8716392; doi:10.3389/fonc.2021.737982)
Supplement: Supplementary Table 1 — Meta-regression analysis of heterogeneity for survival outcomes. [file Table_1.docx]

Supplementary Table 1. Meta-regression analysis of heterogeneity for survival outcomes.

| **Outcomes** | **Variables of regression** | No. of trials | No. of patients | **RRinteraction (95% CI)** | **P-value of regression** | **I^2^** | **Cochrane Q p-value** |
| --- | --- | --- | --- | --- | --- | --- | --- |
| OS | Year | 5 | 5016 | 1.010(0.815-1.250) | 0.9303 | 0.00% | 0.5205 |
|  | Tumor size | 5 | 5016 | 7.303(0.270-197.305) | 0.2372 | 0.00% | 0.8329 |
|  | ER positive rate | 3 | 4886 | 15.133(0.059-3861.963) | 0.3366 | 0.00% | 0.3553 |
|  | Negative lymph node rate | 3 | 4886 | 1.711(0.178-16.468) | 0.6421 | 33.61% | 0.2197 |
|  | Follow up time | 5 | 5016 | 1.025(0.939-1.119) | 0.5760 | 0.00% | 0.5821 |
| DFS | Year | 5 | 5016 | 1.118(0.899-1.390) | 0.3159 | 90.99% | <0.0001 |
|  | Tumor size | 5 | 5016 | 0.041(0.000-10887.870) | 0.6155 | 93.19% | <0.0001 |
|  | ER positive rate | 3 | 4886 | 0.005(0.000-19634175361405.023) | 0.7702 | 97.67% | <0.0001 |
|  | Negative lymph node rate | 3 | 4886 | 0.022(0.000-987.947) | 0.4834 | 97.34% | <0.0001 |
|  | Follow up time | 5 | 5016 | ***1.192(1.037-1.370)*** | ***0.0133*** | 71.03% | 0.0158 |
| LRFS | Year | 5 | 5016 | 1.149(0.913-1.447) | 0.2355 | 79.24% | 0.0023 |
|  | Tumor size | 5 | 5016 | 0.001(0.000-1.450) | 0.0631 | 47.46% | 0.1266 |
|  | ER positive rate | 3 | 4886 | 0.000(0.000-54.907) | 0.1394 | 72.53% | 0.0564 |
|  | Negative lymph node rate | 3 | 4886 | ***0.008(0.001-0.086)*** | ***0.0001*** | 0.00% | 0.3257 |
|  | Follow up time | 5 | 5016 | 1.147(0.963-1.366) | 0.1246 | 70.49% | 0.0172 |
| DMFS | Year | 4 | 4896 | 0.947(0.714-1.256) | 0.7049 | 0.00% | 0.7461 |
|  | Tumor size | 4 | 4896 | 3.048(0.135-68.994) | 0.4838 | 0.00% | 0.8874 |
|  | ER positive rate | 3 | 4826 | 7.548(0.040-1424.196) | 0.4496 | 0.00% | 0.6936 |
|  | Negative lymph node rate | 3 | 4826 | 2.372(0.318-17.709) | 0.3998 | 0.00% | 0.8937 |
|  | Follow up time | 4 | 4896 | 0.976(0.871-1.094) | 0.6795 | 0.00% | 0.7564 |
| CSS | Year | 5 | 5016 | 0.952(0.743-1.219) | 0.6949 | 0.00% | 0.9632 |
|  | Tumor size | 5 | 5016 | 1.887(0.008-424.056) | 0.8182 | 0.00% | 0.9435 |
|  | ER positive rate | 3 | 4886 | 2.231(0.000-44482.417) | 0.8737 | 0.00% | 0.8201 |
|  | Negative lymph node rate | 3 | 4886 | 1.558(0.046-53.130) | 0.8054 | 0.00% | 0.8986 |
|  | Follow up time | 5 | 5016 | 0.977(0.862-1.107) | 0.7141 | 0.00% | 0.9595 |

HRinteraction: interaction effect calculated by meta-regression;

Bold italics indicate statistically significant values (P < 0.05).
